# Vocal Mimicry, and Conspecific Song and Calls, in Female Albert's Lyrebirds (*Menura alberti*)

**DOI:** 10.1002/ece3.72072

**Published:** 2025-08-29

**Authors:** Fiona Backhouse, Justin A. Welbergen, Victoria I. Austin, Anastasia H. Dalziell

**Affiliations:** ^1^ Hawkesbury Institute for the Environment Western Sydney University Penrith New South Wales Australia; ^2^ Gulbali Institute Charles Sturt University, Albury Campus Albury New South Wales Australia; ^3^ Cornell Lab of Ornithology Cornell University Ithaca New York USA

**Keywords:** bird song, female song, lyrebird, nest defence, vocal mimicry

## Abstract

The vocalisations of female songbirds are more complex, widespread, and functionally important than previously thought; yet information is still depauperate compared to that of males. Here we provide the first recordings and analysis of the vocal behaviour of female Albert's lyrebirds, a species in which males are known for elaborate song and dance displays involving vocal mimicry. We document one female Albert's lyrebird vocalising during nest construction and another vocalising at a nest containing a nestling and find that, like males, female Albert's lyrebirds produce vocal mimicry, conspecific song, and alarm calls. However, female repertoires are both distinct from those of the males and used during female‐specific contexts. Our results highlight the potential complexity of vocalisations produced by female songbirds and add to a small but growing list of species in which females produce vocal mimicry.

## Graphical abstract

The vocalisations of female songbirds are more complex, widespread and functionally important than previously thought, yet information is still depauperate compared to that of males. Here we provide the first recordings and analysis of vocal mimicry and conspecific song and calls in the female Albert's lyrebird. We found that female repertoires were complex, distinct from those of males and used in female‐only contexts, highlighting the need to study both males and females to fully understand animal communication.

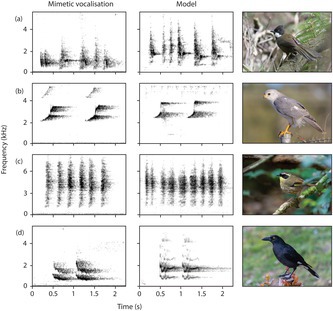

## 1 Introduction

Naturalists have long been captivated by the songs and calls of birds, particularly the elaborate songs of male songbirds. However, until recently, the vocalisations of female songbirds have been overlooked in both natural history accounts and scientific research. We now know that female song is both widespread and ancestral (Odom et al. 2014). Female calls are also widespread and are often female‐specific or female‐typical and used in contexts both similar to and different from those of males (Amy et al. 2018). It is increasingly clear that the evolutionary significance of female vocalisations is far greater than was traditionally assumed. However, the scientific literature is still lacking information on female bird song, likely due to inherent biases about sex roles and because females of many species are less conspicuous than the males and therefore harder to find and observe (Austin et al. 2021; Wu et al. 2025). This has led to calls for more documentation of female bird song (Odom and Benedict 2018; Riebel et al. 2019; Austin et al. 2021).

Female songbirds vocalise in a broad range of contexts (Amy et al. 2018; Odom and Benedict 2018; Austin et al. 2021), including for mate attraction (Langmore et al. 1996; Langmore and Davies 1997), resource or territorial defence (Cooney and Cockburn 1995; Cain et al. 2015; Illes 2015) and nest defence (Dalziell and Welbergen 2016). In many species, individuals can exhibit sex‐specific behaviours, including, but not limited to, males advertising their quality via elaborate displays and females engaging in female‐only parental care. Where such behavioural differences exist, at least some sex‐specificity in vocalisations is also expected (Riebel et al. 2019; Austin et al. 2021; Odom, Cain, et al. 2021). In addition, while ‘song’ is traditionally considered a learnt and complex male vocalisation used during the breeding season, there are ‘innumerable exceptions’ (Catchpole and Slater 2008), and many female songbirds produce complex song‐like vocalisations that are not well understood (Austin et al. 2021). Clearly, a thorough documentation of female songbird vocalisations and the contexts in which they occur (Riebel et al. 2019) will be essential for a more complete understanding of the function of all songbird vocalisations.

While species‐typical songs and calls (‘conspecific vocalisations’) are increasingly documented in female songbirds, females of some species vocally mimic other species or environmental sounds. Vocal mimicry is defined as a vocal sound resembling that of another species, where the acoustic resemblance between the mimic and the heterospecific model induces a behavioural change in the receiver that leads to a selective advantage for the mimic (Dalziell et al. 2015). Mimetic vocalisations are produced by 11%–15% of all songbirds (Goller and Shizuka 2018), yet only a handful of studies have examined mimicry in females (e.g., Dalziell and Welbergen 2016; Dutour et al. 2019; Gammon and Stracey 2022). While some of the most well‐known vocal mimicry is used in mate attraction (e.g., common starling, *Sturnus vulgaris* : Hindmarsh 1984; superb lyrebird, *Menura novaehollandiae* : Zann and Dunstan 2008; northern mockingbird, *Mimus polyglottos* : Gammon 2014), vocal mimicry can also be used in contexts equally relevant to females as to males, such as predation avoidance, parasitism, foraging and competition (Dalziell et al. 2015). Thus, investigating vocal mimicry in females will give a broader perspective on studying the evolution and function of vocal mimicry. Further, given how widespread vocal mimicry is among male songbirds, and the lack of natural history accounts on female vocalisations, it would not be surprising if vocal mimicry is more prevalent in female songbirds than currently assumed.

Here we report both conspecific and mimetic vocalisations in female Albert's lyrebirds ( *Menura alberti* ). Albert's lyrebirds are large oscine passerines found in a restricted area in eastern Australia. They are known for the elaborate mimetic song and dance displays of the males (Robinson and Curtis 1996) and there are now several detailed studies of the males' vocalisations, including conspecific ‘whistle song’ (Backhouse et al. 2021), sequences of highly accurate vocal mimicry termed ‘recital mimicry’ (Putland et al. 2006; Backhouse, Dalziell, Magrath, and Welbergen 2022), and a rhythmic conspecific vocalisation used during a coordinated song and dance display termed ‘gronking song’ (Backhouse, Welbergen, et al. 2024). Each of these male vocalisation types varies considerably between geographic locations (Robinson and Curtis 1996; Backhouse et al. 2021; Backhouse, Dalziell, Magrath, and Welbergen 2022). Albert's lyrebirds share many ecological and behavioural traits with their sister species, superb lyrebirds ( *M. novaehollandiae* ), and female superb lyrebirds produce both conspecific song and calls, as well as vocal mimicry (Dalziell and Welbergen 2016). Female Albert's lyrebirds have been reported to occasionally produce both conspecific song and vocal mimicry (Curtis 1998; Higgins et al. 2001), but no details on their vocalisations have been published. Here, we provide the first formal description of the vocalisations of female Albert's lyrebirds and the contexts in which they occur.

## 2 Methods

### 2.1 Study Species

Albert's lyrebirds are large (~930 g), ground‐dwelling oscine passerines found in a small area of montane subtropical and temperate rainforest and wet sclerophyll forest between 28.89° S and 27.89° S, and 152.36° E and 153.46° E in Bundjalung country, eastern Australia. Male Albert's lyrebirds are territorial and largely solitary, and compete for mates by performing elaborate song and dance displays. Females provide all parental care. Females can be distinguished from males by the lack of ornamented tail feathers and can be identified by their parental behaviour (Higgins et al. 2001).

Very little is known about the breeding behaviour of female Albert's lyrebirds, beyond that they build large, dome‐shaped nests and lay a single egg in the Austral winter (Higgins et al. 2001). The chick stays in the nest for approximately 5 weeks before fledging and is thought to rely on the mother for several months thereafter (based on information on superb lyrebirds, Higgins et al. 2001). Like female superb lyrebirds (Austin et al. 2019) and male Albert's lyrebirds, female Albert's lyrebirds may be territorial (Higgins et al. 2001).

### 2.2 Field Recordings

Field work for this study was undertaken in the breeding seasons (May—August) of 2018 and 2019 as an adjunct to a larger study focused on male Albert's lyrebirds (e.g., Backhouse et al. 2021, 2023). Female Albert's lyrebirds are difficult to find and record; nevertheless, we were able to record two lyrebirds that were clearly female.

In July 2019, we were recording male Albert's lyrebirds at Tamborine (QLD; 27.94° S, 153.20° E) when F.B. opportunistically identified and recorded a female that was first observed singing from a tree. This individual was then followed and recorded for 15 min until the recordist could no longer locate the bird in dense vegetation and rough terrain. This recording was taken using a handheld Sennheiser ME 66/K6 shotgun microphone and a Marantz PMD 661 with a 94 kHz sample rate and 24‐bit depth.

**FIGURE 1:**
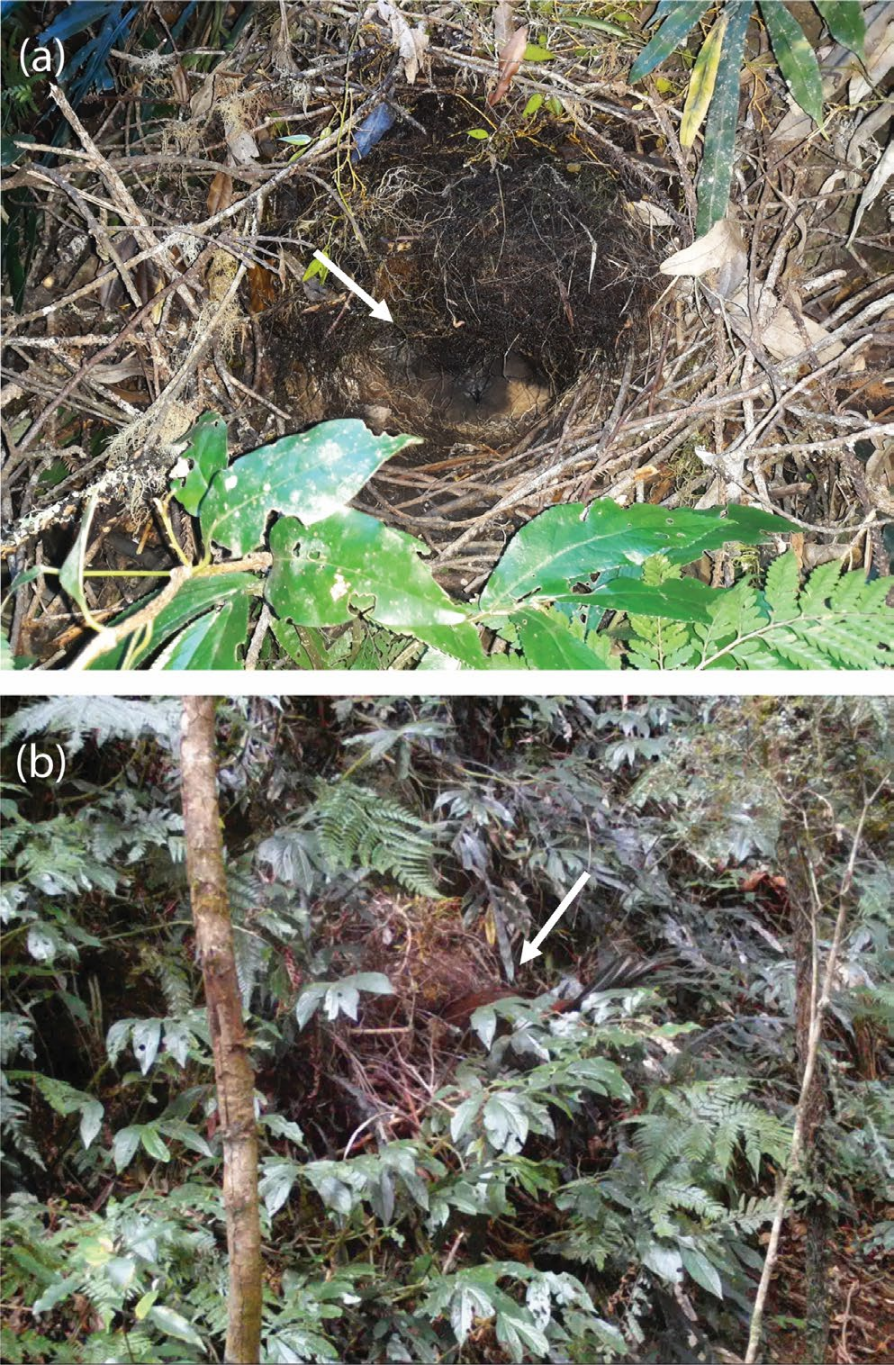
The Albert's lyrebird nest found at Goomburra with (a) the chick visible and (b) the female attending. The chick and the female are each highlighted with a white arrow.

In addition, a concerted nest‐finding effort in August 2018 yielded an active nest at which we could record. Nests were searched for using intensive foraging signs and discarded faecal sacs as cues of nearby active nests (following Maisey et al. 2019). On 28 August 2018 we found a nest in the Goomburra region of Main Range National Park (QLD; 27.97° S, 152.38° E; over 80 km from Tamborine) containing a chick that was estimated to be 4 weeks old (Figure 1), based on comparisons of nestling superb lyrebirds of known ages (A. Maisey, pers. comm.; consistent with Lill 1979). We placed a motion sensing camera (Stealth Cam DS4K) and an autonomous sound recorder (SM4: Wildlife Acoustics) at the nest. The SM4 was set to record from 1 h before sunrise to 3 h after sunrise, and for 2 h before sunset to 1 h after sunset, at 12 dB gain and 48 kHz sampling rate. Female superb lyrebirds often sing when leaving the nest each morning after incubating or staying with the chick overnight (V.I.A., unpublished data). We therefore focussed our observations on the first 2 h of recording time each morning, checking further recordings where continuous vocalisations were apparent. The SM4 recorded from 29 August 2018 to 20 September 2018. SM4 recordings suggested that the chick fledged on the 15th of September. There were no vocalisations at or close to the nest after this date.

### 2.3 Female Identification

Vocalisations of male Albert's lyrebirds are easily identifiable due to their highly regular syntactical structure (Backhouse, Dalziell, Magrath, and Welbergen 2022) and loyalty to set display platforms (Backhouse, Mirando, et al. 2024; Backhouse, Welbergen, et al. 2024). Far less is known about female vocal behaviours. Adult female lyrebirds are not easily distinguishable from juvenile males, a general problem that may have led to the underestimation of female song in lyrebirds and other species (Dalziell and Welbergen 2016; Odom and Benedict 2018).

The females in the recordings used here were identified with high certainty. The lyrebird recorded at Tamborine was identified as a breeding female by her plain tail and as she was first observed holding nesting material (tree fern fibres) in her bill, which she dropped soon after observing the recordist. This is a uniquely female behaviour as male Albert's lyrebirds are not involved in nest building (Higgins et al. 2001). Vocalisations of this female in the recording taken were then identified as lyrebird vocalisations (rather than nearby heterospecifics) with high confidence as she was directly observed during the recording. The lyrebird recorded at Goomburra was identified as a breeding female due to her repeated visits to the nest, confirmed via camera trap footage (Figure 1; Video S1). Nest visits captured on camera confirmed broadband rustling sounds when the female entered the nest and wingbeats and further rustling sounds when leaving the nest, and in‐nest vocalisations of the female and the chick. To ensure that all vocalisations reported here belong to the nesting female and not to any other lyrebird nearby, we only report vocalisations that were part of a sequence starting or ending within 3 min of a nest visit, identified through sounds consistent with the female entering and leaving the nest. Mimetic vocalisations were assumed to be produced by the female, rather than a nearby heterospecific, if they were interspersed with and at a qualitatively similar amplitude to conspecific alarm calls.

### 2.4 Acoustic Analysis

We classified vocalisations into four mutually exclusive categories of ‘vocal units’: (i) ‘mimetic’, (ii) ‘whistle song’, (iii) ‘alarm calls’ or (iv) ‘in‐nest vocalisations’. Individual vocal units were defined as a production of sound of the same type (e.g., alarm call, whistle song, type of mimicry) with a temporal gap of < 1 s between units (following Backhouse, Dalziell, Magrath, and Welbergen 2022; Backhouse et al. 2023). A vocal unit could be one or more elements, with an element defined as a discrete, continuous sound (Odom, Araya‐Salas, et al. 2021). Mimetic units were defined by model species and by model vocalisation type (alarm call, non‐alarm call, song) using comparisons with recordings of model species archived on Macaulay Library (Cornell Lab of Ornithology 2025) and Xeno‐canto (Xeno‐canto foundation 2025) and information on vocalisation function in Handbook of Australian, New Zealand & Antarctic Birds (HANZAB; BirdLife Australia 2023). Vocal units that could not be attributed to mimicry of any species and were acoustically similar to known female superb lyrebird alarm calls (Dalziell and Welbergen 2016; Austin 2022) were identified as conspecific alarm calls. Conspecific whistle songs were identified through acoustic similarities with male Albert's lyrebird whistle songs (e.g., Backhouse et al. 2021). In‐nest vocalisations were identified based on similarities with descriptions of in‐nest calls recorded in the superb lyrebird (Guppy et al. 2021).

Recordings were viewed as spectrograms in Raven Pro 64 bit 1.6.5 with a ‘Hann’ display type, the window set at Fast Fourier Transform 1050, and a grid resolution of 21.9 ms and 65.8 Hz. We measured female conspecific vocalisations using selection boxes in Raven Pro. For whistle songs, we measured peak frequency, frequency bandwidth and duration of vocal units; and for conspecific alarm calls, we measured peak frequency and frequency bandwidth of vocal units, but not duration as this varied considerably with the number of repeated elements. For in‐nest vocalisations, we measured peak frequency and duration of individual elements, but not frequency bandwidth as this varied considerably with the strength of the harmonics. Acoustic measurements were only taken from vocal units with a high enough signal‐to‐noise ratio to visually identify individual elements. We made preliminary and qualitative comparisons of our recordings of two female Albert's lyrebirds with previously collected data on male Albert's lyrebirds from the same sites and an additional five populations [male whistle songs (*N*_males_ = 32): Backhouse, Dalziell, Magrath, Rice, et al. (2022); male recital mimicry (*N*_males_ = 35): Backhouse, Welbergen, Magrath, and Dalziell (2022)].

## 3 Results

### 3.1 Female Observations

The Tamborine female was observed giving alarm calls and vocal mimicry from high in a tree before she flew to the ground and foraged while she continued vocalising. In 14 min 30 s of recording time, this female produced a total of 64 vocal items comprising 32 conspecific alarm calls interspersed with 24 vocal mimetic units and five songs reminiscent of but different to the standard whistle songs of the males in the same study area. Just 3 of 64 (0.05%) vocal items were not identified.

The SM4 placed near the nest at Goomburra recorded vocalisations attributable to the female both in the nest and in the surrounding area. Most vocalisations recorded were in‐nest contact calls of the female and begging calls of her chick during regular nest visits to feed the chick and remove faecal sacs. From the evening of the 29th of August until the morning of the 6th of September, the female spent the night in the nest and produced songs resembling the whistle songs of males upon leaving the nest just before sunrise. Occasional isolated whistle songs were recorded near the nest on some mornings thereafter, including following nest visits, though we did not include these songs in the analysis due to lower certainty of the singer's identity.

On most mornings, the Goomburra female was silent outside nest visits. However, on the morning of the 30th of August, the SM4 recorded frequent alarm calls and vocal mimicry leading up to and following a nest visit (Audio S1). Across 26 min of recorded vocal mimicry, we identified mimicry of six bird species and one mammal species. Most mimicry was either of eastern whipbirds ( *Psophodes olivaceus* ; 102 of 176 mimetic units, 56.0%) or grey goshawks (*Tachyspiza novaehollandiae*; 42 of 176 mimetic units, 23.9%). Alarm calls and mimicry in recordings thereafter were rare.

### 3.2 Vocal Mimicry

**FIGURE 2:**
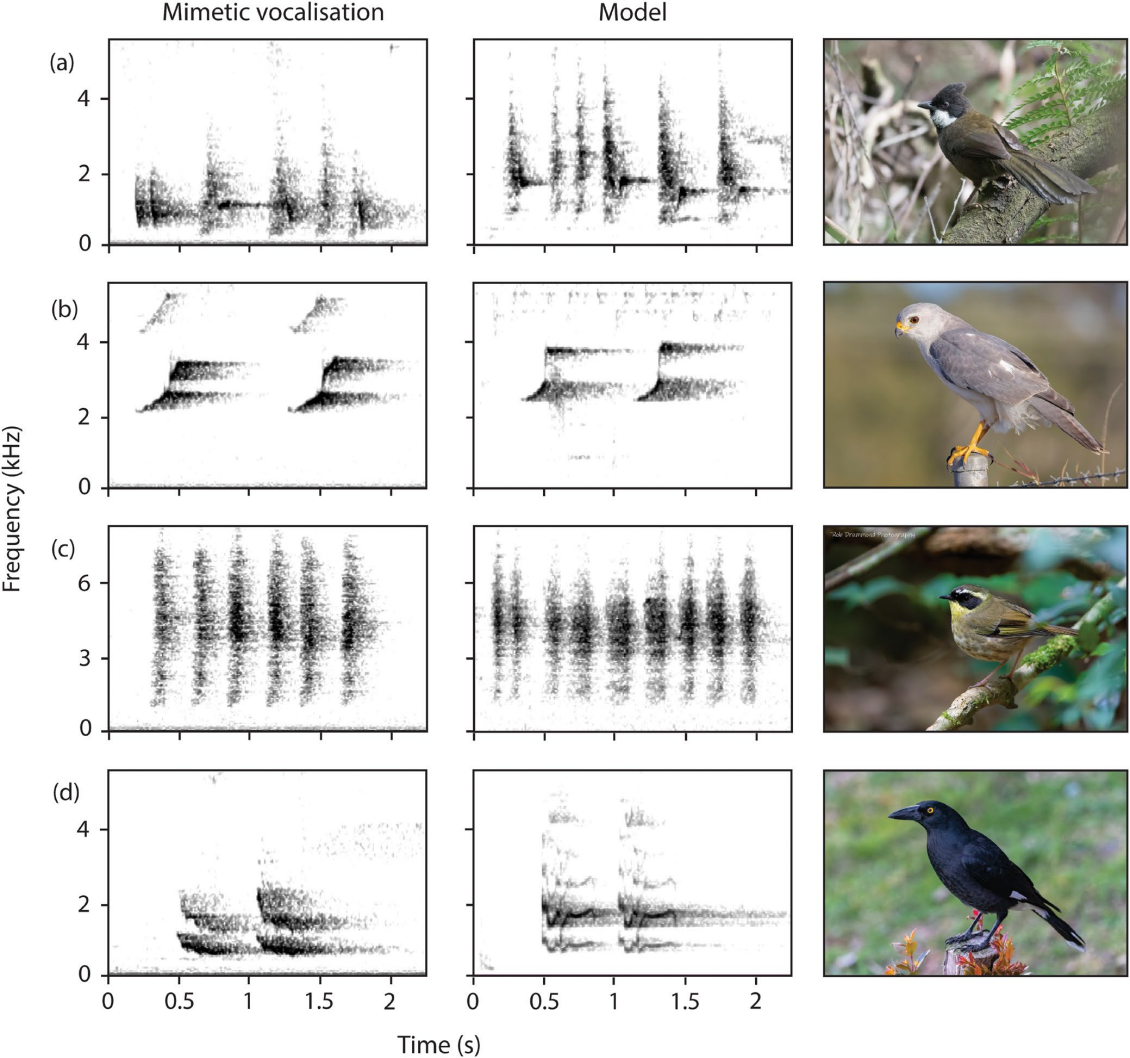
Four common mimetic vocalisations produced by the female Albert's lyrebirds in this study (left), the corresponding vocalisations produced by the models (centre) and the model species (right). (a) Eastern whipbird contact call ( *Psophodes olivaceus* ); (b) grey goshawk call (*Tachyspiza novaehollandiae*); (c) yellow‐throated scrubwren alarm call (*Neosericornis citreogularis*); (d) pied currawong call ( *Strepera graculina* ). Audio recordings of the mimetic vocalisations are available in Audios S2–S5. Model recordings for (a–c) were obtained from *Xeno‐canto* (CC) (Anderson 2012, 2014, 2018) and the model recording for (d) was reproduced with permission from *Macaulay Library* (ML229695: Powys 2008). Model photos reproduced with permission from John Daniels (eastern whipbird), Richard Simmonds (grey goshawk; www.rsnaturephotography.com), Rob Drummond (yellow‐throated scrubwren) and Jill Duncan (pied currawong).

Both females mimicked the songs, contact calls and alarm calls of heterospecifics harmless to lyrebirds and also mimicked the calls of species known or likely to depredate superb lyrebird nests (Table 1; V.I.A., unpublished data). The two Albert's lyrebird females largely differed in their mimetic repertoires, with only eastern whipbirds and grey goshawks mimicked by both females (Table 1), though most model species have been observed or are expected to occur at both sites (Backhouse et al. 2023). The mimicry was identified with high confidence and had qualitatively close resemblance to the model calls (e.g., Figure 2). Most of the species mimicked by the recorded females were also mimicked by male Albert's lyrebirds recorded previously (see Backhouse et al. 2021; Backhouse, Dalziell, Magrath, and Welbergen 2022; Backhouse, Welbergen, Magrath, and Dalziell 2022), with the exception of eastern whipbirds, pied currawongs and Australian magpies (Table 1). However, there were also species and vocalisation types that were mimicked by females in this study that were not recorded in males at the same site, or were only occasionally mimicked by males.

**TABLE 1:** The species and vocalisation types mimicked by Tamborine and/or Goomburra females in this study (*N*_females_ = 2), and the presence of this mimicry in the repertoires of males from Tamborine, Goomburra or ‘other populations only’ [male data from Backhouse, Dalziell, Magrath, Rice, et al. (2022; *N*_males_ = 32 from *N*_populations_ = 6) and Backhouse, Welbergen, Magrath, and Dalziell (2022; *N*_males_ = 35 from *N*_populations_ = 7)].

Model species	Vocalisation	Female mimicry	Male mimicry
Eastern whipbird ( *Psophodes olivaceus* )	Contact call	Both females	No
	Whipcrack duet	Tamborine	No
Grey goshawk (*Tachyspiza novaehollandiae*)	Call	Both females	Goomburra
Eastern yellow robin ( *Eopsaltria australis* )	Song	Tamborine	Goomburra
Crimson rosella ( *Platycercus elegans* )	Alarm call	Tamborine	Both populations
Satin bowerbird ( *Ptilonorhynchus violaceus* )	Display song	Tamborine	Both populations
Unknown	Wingbeats	Tamborine	Both populations
Unknown	Taps/rattles	Tamborine	Both populations
Yellow‐throated scrubwren (*Neosericornis citreogularis*)	Alarm call	Goomburra	Other populations only
Lewin's honeyeater ( *Meliphaga lewinii* )	Alarm call	Goomburra	Other populations only^a^
Pied currawong ( *Strepera graculina* )	Call	Goomburra	No
Australian magpie ( *Gymnorhina tibicen* )	Alarm call	Tamborine	No
Short‐eared possum ( *Trichosurus caninus* )	Call	Goomburra	Both populations^b^
Wedge‐tailed eagle ( *Aquila audax* )	Call	Goomburra	Other populations only^b^

*Note:* Species are ordered by their popularity in the repertoire of either female.

^a^ Lewin's honeyeater song mimicked by males at Goomburra, alarm calls mimicked elsewhere.

^b^ Rarely mimicked.

### 3.3 Conspecific Alarm Calls

Both female Albert's lyrebirds produced low‐frequency calls acoustically similar to calls produced by female superb lyrebirds and referred to as alarm calls (Higgins et al. 2001; Dalziell and Welbergen 2016). We identified four variants (Table 2A; Audios S6–S9); a low ‘growl’ repeated 1–4 times in quick succession; a two to three element call beginning with a growl and ending in one or two short, higher frequency elements, similar to the two‐note ‘aw‐kok’ in Dalziell and Welbergen (2016; termed ‘aw‐kok’ here for consistency); a low growl ending in a short frequency sweep (‘growl‐squeak’); and a short frequency sweep without the preceding growl (‘squeak’).

**TABLE 2:** Summary acoustic measurements for the female conspecific vocalisations recorded for this study.

A: Alarm calls					
Alarm call name	Superb lyrebird analogue	*N* recorded (*N* measured)	Peak frequency (Hz)	Frequency bandwidth (Hz)	Spectrogram
Growl^a^	Alarm B^b^	116 (89)	742 ± 120	1485 ± 297	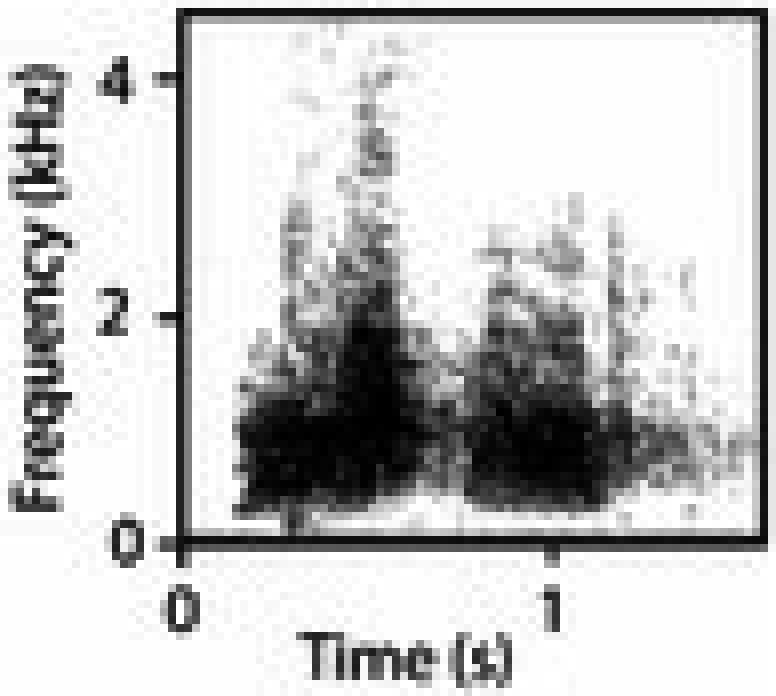
Aw‐kok	Aw‐kok^b^	40 (30)	923 ± 396	1926 ± 119	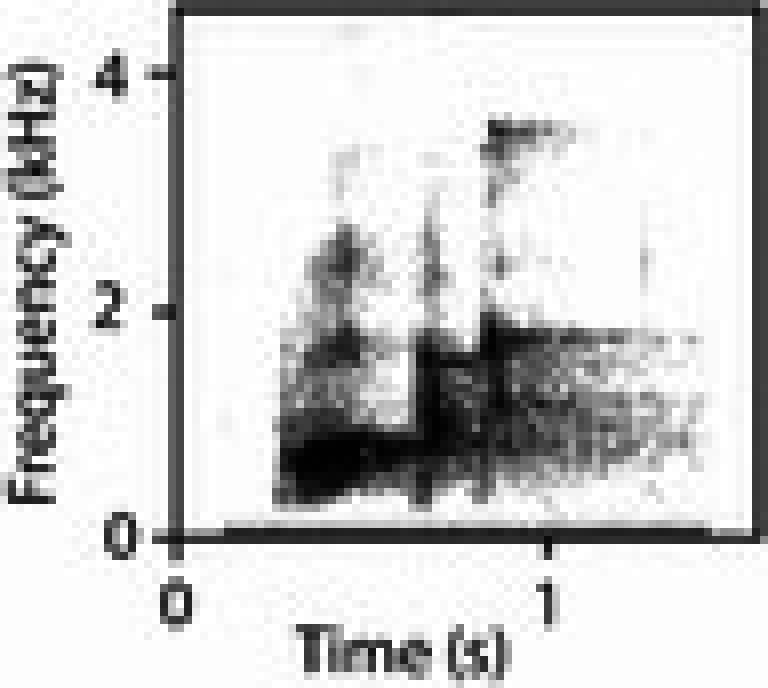
Growl‐squeak	Un‐named vocalisation^c^	36 (34)	895 ± 397	2552 ± 155	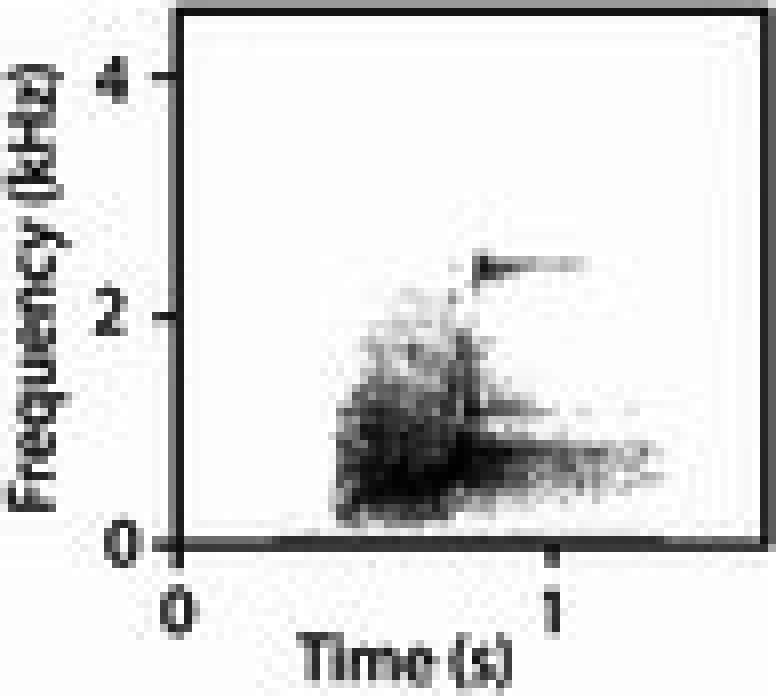
Squeak	Un‐named vocalisation^c^	13 (12)	1111 ± 109	2713 ± 211	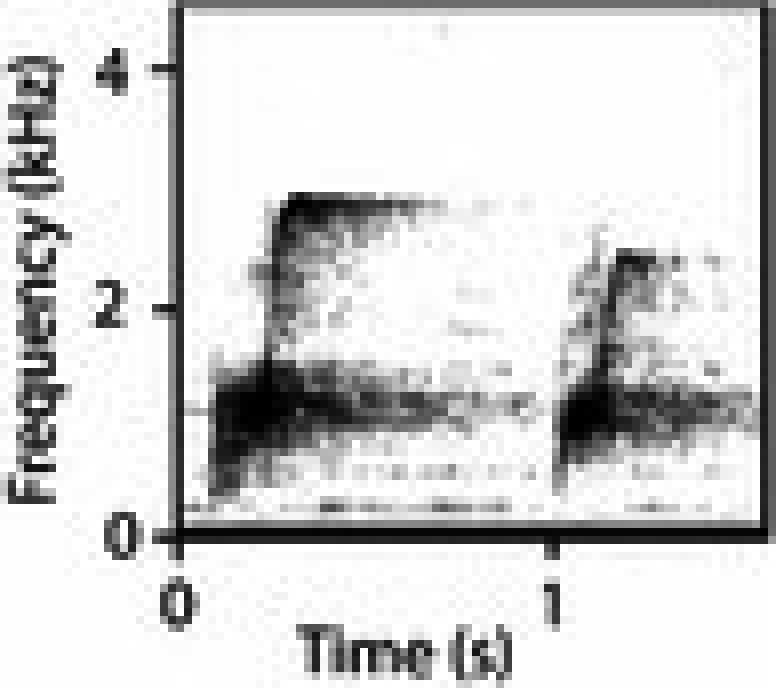

B: Whistle songs						
Singer identity	Song type	*N* recorded (*N* measured)	Peak frequency (Hz)	Frequency bandwidth (Hz)	Duration (s)	Number of elements
Tamborine female	All	5 (5)	1688 ± 435	2874 ± 175	4.91 ± 2.72	12.4 ± 7.09
Goomburra female	‘Male‐type’	34 (30)	2267 ± 429	2972 ± 102	4.67 ± 0.70	12.0 ± 1.53
	‘Atypical’	22 (20)	2040 ± 579	2970 ± 143	2.64 ± 0.62	7.64 ± 2.13

C: In‐nest contact calls			
Singer identity	*N* measured	Peak frequency (Hz)	Duration (s)
Goomburra female	13 elements from one nest visit	386 ± 192	0.18 ± 0.04

*Note:* Acoustic measurements are mean ± SD. Note that some different measurements were taken for each vocalisation type. Example audio recordings of each vocalisation are available in Audios S6–S14.

^a^ The female recorded at Tamborine only produced the ‘growl’ alarm.

^b^ Dalziell and Welbergen (2016).

^c^ V.I.A. (unpublished data).

### 3.4 Conspecific Song

**FIGURE 3:**
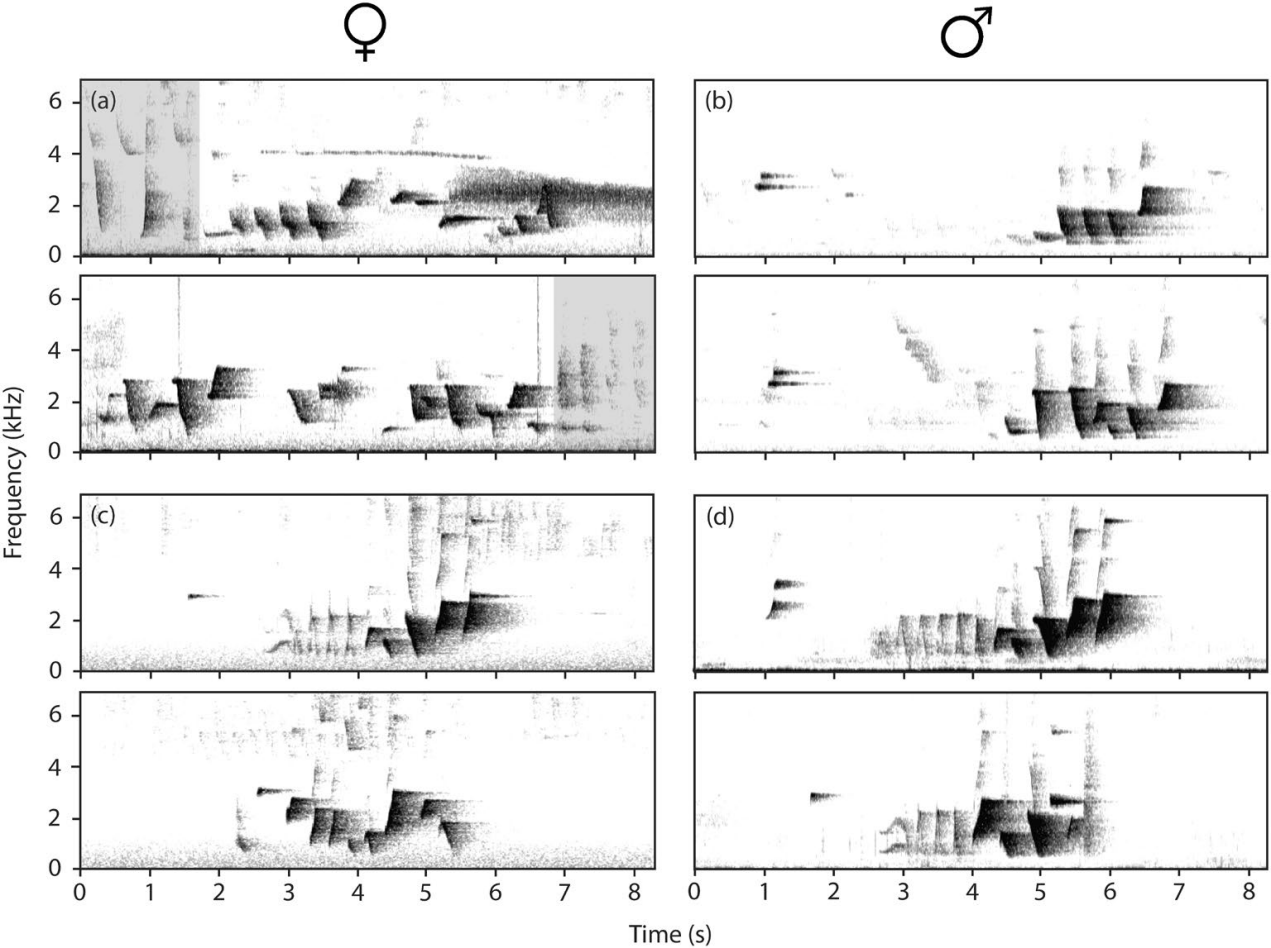
Whistle songs of female (left) and male (right) Albert's lyrebirds. (a) Two whistle songs from the female lyrebird at Tamborine. Mimetic units preceding and following the whistle songs are greyed out. This recording included interference from an eastern spinebill (the line at 4 kHz) and a Lewin's honeyeater (3 kHz from 5 s). (b) Two whistle songs from a male lyrebird at Tamborine, recorded on the same day as the female. Note the clear introductory element in each, which was absent in all female whistle songs at this site. (c) Two whistle songs from the female lyrebird at Goomburra. The top whistle song is an exemplar of the ‘male‐type’ whistle song, and the bottom is an exemplar of a whistle song type not recorded in the males. (d) Two typical whistle songs of a male lyrebird at Goomburra, recorded 27 June 2018. Audio recordings of the female whistle songs are available in Audios S10–S13.

The Tamborine female sang five ‘whistle songs’ of 5–22 elements long (Table 2B). These whistle songs were on average 4.3 elements longer than the average whistle song of the males in the same population (mean 8.10 ± 0.84 SD elements, *n*_songs_ = 134 from *n*_males_ = 5; data from Backhouse, Dalziell, Magrath, Rice, et al. 2022). Four out of five whistle songs included a sequence of elements that superficially resembled the whistle song of the males but contained further element sequences that were not recorded in the males at Tamborine (Figure 3). No whistle song had an introductory element, a consistent component of the male whistle song throughout the species' range (Backhouse et al. 2021).

From the 30th of August to the 6th of September 2018, the nesting Goomburra female gave 1–15 whistle songs within 10 min of leaving the nest each morning (*n* = 56 total; Table 2B). 34 of the whistle songs (60.7%) had a close visible and audible resemblance to the regular whistle songs of the local males (Figure 3) and contained 8–15 elements (local male songs contained mean 11.85 ± 1.63 SD elements, *n*_songs_ = 135 from *n*_males_ = 6; data from Backhouse, Dalziell, Magrath, Rice, et al. 2022). The remaining 22 (39.2%) songs resembled atypical whistle songs that we have recorded before but could not reliably attribute to male or female lyrebirds, and contained 5–11 elements. All but one ‘male‐type’ whistle song (97.1%) contained an introductory element, whereas none of the ‘atypical’ whistle songs contained an introductory element. The Goomburra female sang both ‘male‐type’ and ‘atypical’ whistle song types after leaving the nest on three of seven mornings analysed, whereas for two mornings she sang only the ‘male‐type’ whistle song, and the remaining two mornings she sang only the ‘atypical’ whistle song.

### 3.5 In‐Nest Vocalisations

**FIGURE 4:**
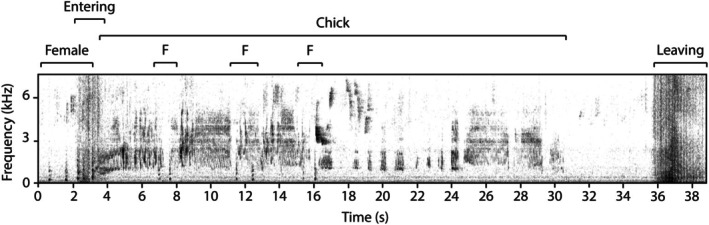
A typical sequence of the vocalisations produced by the female (‘F’) and her chick during nest visits. The female produced contact calls (short vocalisations < 1.5 kHz) prior to and after entering the nest (rustling at 2 s), upon which the chick commenced begging calls until soon before the female left the nest (wingbeats at 36 s). The two loud elements at 16 s are from a white‐throated treecreeper. The audio recording of this nest visit is available in Audio S14.

Each time the Goomburra female attended the nest, she and her nestling both vocalised. The female produced short, low frequency, quiet contact calls with prominent harmonics (Table 2C). These contact calls resemble calls made prior to feeding in the superb lyrebird described by Guppy et al. (2021). Upon the female entering the nest, the female's contact calls were accompanied by quiet, broadband, trill‐like warbles of the chick that continued until the female left the nest (Figure 4; Audio S14).

## 4 Discussion

This study, describing for the first time the vocal mimicry, conspecific song and calls in female Albert's lyrebirds, confirms previous reports that female Albert's lyrebirds possess their own complex vocal repertoires. We document in‐nest vocalisations and alarm calls that may be female‐specific and found that female Albert's lyrebirds have conspecific whistle songs and are capable of mimicking a wide range of heterospecific models. While this study is limited to just two individuals, these results help us to form hypotheses to test in future work. Our observations highlight the need to study both male and female birds to gain a thorough overview and understanding of a species' vocal repertoire and its functions.

The focal female from Tamborine and the focal female from Goomburra (80 km away) both produced vocal mimicry during different phases of nesting: while building a nest and while caring for a nestling. In addition, the mimicry of both females was interspersed with alarm calls, suggesting that the mimicry may have been used in an alarming context. While vocal mimicry has rarely been documented in female birds, a number of studies have found vocal mimicry used by females during capture, nest disturbance or in the presence of predators (Chu 2001; Igic and Magrath 2013; Møller et al. 2021), including in superb lyrebirds (Dalziell and Welbergen 2016; Austin et al. 2025, in review). Indeed, like female superb lyrebirds (Dalziell and Welbergen 2016; Austin et al. 2025, in review), many of the vocalisations mimicked by the female Albert's lyrebirds could be associated with alarm, either as alarm calls of harmless conspecifics or the calls of potential predators. In contrast, male Albert's lyrebirds mimic a greater range of non‐alarm calls and songs of harmless conspecifics (Backhouse et al. 2023). Furthermore, almost half of the mimicry produced by female Albert's lyrebirds appears to be either absent or rare in the repertoires of male Albert's lyrebirds (Backhouse et al. 2021, 2023). Interestingly, 8 of the 11 species mimicked here were also mimicked by female superb lyrebirds in either the Blue Mountains (NSW, over 1000 km away; Dalziell and Welbergen 2016) or Sherbrooke Forest (VIC; Austin et al. 2025, in review). Further recordings of females of both species and of males outside the display context are required to formally test repertoire similarities.

Only two heterospecifics were mimicked by both female Albert's lyrebirds, and the mimetic repertoires of each female did not reflect the mimetic repertoires of the local males. The differences we found may be driven by different functions for the mimicry relating to their sexually dimorphic behaviour; females may mimic any vocalisation that could aid in‐nest defence, whereas males may mimic vocalisations that highlight their vocal abilities. Female superb lyrebirds are similarly highly variable in repertoire content, even within an individual female when recorded across years (Austin et al. 2025, in review). On the other hand, the socially transmitted mimetic repertoires of male Albert's lyrebirds are highly stereotyped within individuals and populations (Backhouse, Dalziell, Magrath, and Welbergen 2022). Without further recordings of females from each site, we are unable to determine if, like males, female Albert's lyrebirds exhibit local cultures in their mimetic repertoires or if they are highly variable like female superb lyrebirds. Further recordings would also help to determine which species and vocalisations are most commonly mimicked by female Albert's lyrebirds and why they might be chosen.

Both female Albert's lyrebirds produced low frequency, guttural alarm calls that were similar to those produced by female superb lyrebirds but distinct from the classic ‘scream’ alarm calls recorded in male Albert's lyrebirds. Low‐frequency alarm calls have not been described in male Albert's lyrebirds, nor have we recorded them. This suggests that alarm calls may be sex‐specific or context‐dependent. Both male and female Albert's lyrebirds have been observed giving a ‘scream’ alarm when startled (F.B., pers. obs.) or flushed from the nest (Higgins et al. 2001), with the males fleeing while alarming (F.B., pers. obs.). The female guttural alarm calls recorded during this study were produced continually and without immediately fleeing. For males, predators present a danger only to themselves, and so fleeing and avoiding further attention may be the best strategy. For nesting females, predators are a danger to both the female and her young, and so the female Albert's lyrebirds alarm calls documented here may be both sex‐specific and context‐dependent and a response to an ongoing threat. Context‐dependent alarm calls have been documented in a number of bird species, in both conspecific alarm calls (e.g., Platzen and Magrath 2005; Welbergen and Davies 2008; Farrow et al. 2017) and mimetic alarm calls (e.g., Igic and Magrath 2014; Dalziell and Welbergen 2016). It therefore follows that when there are female‐specific contexts, such as nest defence, there are likely also female‐specific alarm calls (Amy et al. 2018; Austin et al. 2021).

Both female Albert's lyrebirds produced conspecific whistle songs that were sometimes highly similar to the whistle song of the local males and sometimes distinct. The whistle songs produced by the Tamborine female were longer and had a greater variety of element types and were also likely produced during an alarming context (i.e., after seeing the recordist). The whistle songs produced by the Goomburra female (recorded in the absence of humans) were either directly comparable to the male whistle songs, or were a different form that was a similar duration or shorter. These Goomburra female whistle songs were sung after first leaving the nest and in the absence of any alarm calls or mimicry and so may signal territory ownership. The whistle songs of male Albert's lyrebirds appear to be used in an advertisement context, though it is unclear whether they are targeted at females, neighbouring males or both (Robinson and Curtis 1996, pers. obs.). In a third population (Lamington National Park) we have observed male Albert's lyrebirds occasionally singing modified, more complex versions of their whistle songs during antagonistic territorial interactions (unpublished data, recorded 2019). Additionally, female superb lyrebirds may use whistle songs in both nest defence and territorial defence (Dalziell and Welbergen 2016), indicating that similar vocalisations may be used in different contexts (e.g., Mennill and Vehrencamp 2008; Wheatcroft 2015). We suggest that across male and female Albert's lyrebirds there may be multiple forms of whistle songs used in different contexts; ‘simple’, stereotyped forms for territory or mate advertisement (e.g., Goomburra female) and more complex forms for agonistic interactions (e.g., Tamborine female).

The two female Albert's lyrebirds recorded for this study demonstrate a rich vocal repertoire, but further work is needed to understand the function and evolution of these vocalisations, and how female vocal repertoires differ from male repertoires. Further, recordings of both females were taken during contexts associated with nesting, and we still lack information on female Albert's lyrebird vocalisations outside these contexts. Much of the vocal repertoires recorded here appears to be unique to females, as several mimetic and non‐mimetic vocalisations were absent from our extensive dataset of male vocalisations. However, broad vocal categories (e.g., whistle songs, vocal mimicry) appear to be shared between males and females but used in different contexts. Such sex‐specificity in the use of shared vocalisation types may be found in other species, particularly those with conspicuous vocal mimicry (e.g., mockingbirds: Gammon and Stracey 2022), suggesting complex evolutionary and ontological trajectories of vocal behaviours in these species. Our research adds to the small but growing body of evidence that vocalisations in female birds are complex, varied and functionally important; and highlights the need to further investigate female vocalisations to enhance our understanding of the communication systems of birds.

## Author Contributions

**Fiona Backhouse:** conceptualization (lead), funding acquisition (supporting), investigation (lead), writing – original draft (lead), writing – review and editing (equal). **Justin A. Welbergen:** conceptualization (supporting), funding acquisition (equal), investigation (supporting), writing – review and editing (equal). **Victoria I. Austin:** conceptualization (supporting), investigation (supporting), writing – review and editing (equal). **Anastasia H. Dalziell:** conceptualization (supporting), funding acquisition (equal), investigation (supporting), writing – review and editing (equal).

## Conflicts of Interest

The authors declare no conflicts of interest.

## Supporting information


**Audio S1.** An example of the female Albert's lyrebird at Goomburra mimicking the contact call of an eastern whipbird (*Psophodes olivaceus*).
**Audio S2**. An example of the female Albert's lyrebird at Goomburra mimicking the call of a grey goshawk (*Tachyspiza novaehollandiae*).
**Audio S3**. An example of the female Albert's lyrebird at Goomburra mimicking the alarm call of a yellow‐throated scrubwren (*Neosericornis citreogularis*).
**Audio S4**. An example of the female Albert's lyrebird at Goomburra mimicking the call of a pied currawong (*Strepera graculina*).
**Audio S5**. The ‘growl’ alarm of a female Albert's lyrebird, recorded at Goomburra.
**Audio S6**. The ‘aw‐kok’ alarm of a female Albert's lyrebird, recorded at Goomburra.
**Audio S7**. The ‘growl‐squeak’ alarm of a female Albert's lyrebird, recorded at Goomburra.
**Audio S8**. The ‘squeak’ alarm of a female Albert's lyrebird, recorded at Goomburra.
**Audio S9‐S10**. Example whistle songs of the female Albert's lyrebird recorded at Tamborine. These whistle songs correspond to Figure 3a in the main text.
**Audio S11‐S12**. Example whistle songs of the female Albert's lyrebird recorded at Goomburra. These whistle songs correspond to Figure 3c in the main text.
**Audio S13**. The contact calls of a female Albert's lyrebird and her nestling recorded during a nest visit. This recording corresponds to Figure 4 in the main text.

**Video S1:** A female Albert's lyrebird visiting her nest to feed the nestling and remove faecal sacs. Filmed at Goomburra using a motion‐sensing trail camera on 2 September 2018.

Audio S1‐S13
Video S1

## Data Availability

Data associated with this paper have been supplied as Supporting Information S1.
