# Supplementary material for: Vocal Mimicry, and Conspecific Song and Calls, in Female Albert's Lyrebirds (Menura alberti)
Source: Ecol Evol. 2025 Aug 29;15(9):e72072. doi: 10.1002/ece3.72072 (PMC12397497; doi:10.1002/ece3.72072)
Supplement: Supplementary file 3 — Audio S1‐S13 Video S1 [file ECE3-15-e72072-s001.docx]

**Supplementary audio and video captions for Backhouse et al. (2025). Vocal mimicry, and conspecific song and calls, in female Albert's lyrebirds (*Menura alberti*). *Ecology and Evolution*.**

**Audio S1.** An excerpt of the alarm calls and mimicry produced by the female Albert’s lyrebird at Goomburra.

**Audio S2.** An example of the female Albert’s lyrebird at Goomburra mimicking the contact call of an eastern whipbird (*Psophodes olivaceus*).

**Audio S3.** An example of the female Albert’s lyrebird at Goomburra mimicking the call of a grey goshawk (*Tachyspiza novaehollandiae*).

**Audio S4.** An example of the female Albert’s lyrebird at Goomburra mimicking the alarm call of a yellow-throated scrubwren (*Neosericornis citreogularis*).

**Audio S5.** An example of the female Albert’s lyrebird at Goomburra mimicking the call of a pied currawong (*Strepera graculina*).

**Audio S6.** The ‘growl’ alarm of a female Albert’s lyrebird, recorded at Goomburra.

**Audio S7.** The ‘aw-kok’ alarm of a female Albert’s lyrebird, recorded at Goomburra.

**Audio S8.** The ‘growl-squeak’ alarm of a female Albert’s lyrebird, recorded at Goomburra.

**Audio S9-S10.** The ‘squeak’ alarm of a female Albert’s lyrebird, recorded at Goomburra.

**Audio S11-S12.** Example whistle songs of the female Albert’s lyrebird recorded at Tamborine. These whistle songs correspond to Figure 3a in the main text.

**Audio S13.** Example whistle songs of the female Albert’s lyrebird recorded at Goomburra. These whistle songs correspond to Figure 3c in the main text.

**Audio S14.** The contact calls of a female Albert’s lyrebird and her nestling recorded during a nest visit. This recording corresponds to Figure 4 in the main text.

**Video S1.** A female Albert’s lyrebird visiting her nest to feed the nestling and remove faecal sacs. Filmed at Goomburra using a motion-sensing trail camera on 2 September 2018.
